# Carleman linearization and normal forms for differential systems with quasi-periodic coefficients

**DOI:** 10.1186/s40064-016-3015-6

**Published:** 2016-08-15

**Authors:** Sergey V. Chermnykh

**Affiliations:** Compulsory Health Insurance Fund of St.Petersburg, Moskovskii pr. 120, St.Petersburg, Russian Federation 196084

**Keywords:** Poincaré normal form, Carleman linearization, General perturbation theory, Primary 34A34, Secondary 34A35

## Abstract

We study the matrix representation of Poincaré normalization using the Carleman linearization technique for non-autonomous differential systems with quasi-periodic coefficients. We provide a rigorous proof of the validity of the matrix representation of the normalization and obtain a recursive algorithm for computing the normalizing transformation and the normal form of the differential systems. The algorithm provides explicit formulas for the coefficients of the normal form and the corresponding transformation.

## Background

The main aim of this paper is to develop a recursive algorithm for constructing transformations to the Poincaré normal form for non-autonomous differential systems with quasi-periodic coefficients (Arnold 1983), suitable for performing on a computer. The algorithm is based on the Carleman linearization technique (Carleman 1932).

Several applications of Carleman linearization have been presented up until now. Bellman (1961) used Carleman linearization to obtain approximate solutions to nonlinear systems. Babadzanjanz (1978) studied the existence of continuations and representation of the solutions in celestial mechanics. Steeb and Wilhelm (1980) and Kowalski and Steeb (1991) studied nonlinear dynamical systems and generalizations of Carleman linearization. Carleman linearization technique has been used in a series of applications in the field of control theory, for example, to controlability and observability of infinite-dimentional linear dynamical systems (Mozyrska and Bartosiewicz 2006, 2008) and to stochastic systems (Germani et al. 2007).

The connection between Carleman linearization and the Poincaré-Dulac normal form for autonomous differential systems has been the subject of work by Tsiligiannis and Lyberatos (1989), Della Dora and Stolovitch (1991). The refinement of classical normal form for dynamical systems was proposed by Chen and Della Dora (1999, 2000).

In this paper, we apply Carleman linearization to the problem of constructing the Poincaré normal form for non-autonomous differential equations with quasi-periodic coefficients, as proposed in Chermnykh (1987).

Poincaré normalization for non-autonomous differential systems with quasi-periodic coefficients is used, for example, in celestial mechanics to construct the GPT-method of general planetary theory (Brumberg 1970; Brumberg and Chapront 1973) and GPT-compatible methods of the general Earth’s rotation theory (Brumberg and Ivanova 2011) and the Moon’s motion theory (Ivanova 2014). Planetary theories have been historically developed to provide ephemerides of planetary bodies; reviews of planetary theories can be found in Seidelmann (1993) and Kholshevnikov and Kuznetsov (2007).

The first step of GPT-method is to reduce the differential system in the vicinity of unperturbed motion by a proper choice of coordinates to the form

1 $$\dfrac{d}{dt}\,{\mathbf {X}}={\mathbf {f}}\left( {\mathbf {X}},t\right) ,$$

where the components of vector **f** are holomorphic functions with respect to the components of vector ${\mathbf {X}}\in {\mathbb {C}}^{\nu }$; **f** depends on *t* by means of quasi-periodic functions; ${\mathbf {f}}\left( {\mathbf {0}},t\right) ={\mathbf {0}}$; the Jacobi matrix $\left. \left( \partial {\mathbf {f}}/\partial {\mathbf {X}}\right) \right| _{{\mathbf {X}}=0}$ is of Jordan form (even diagonal) with purely imaginary eigenvalues.

The second step of the GPT-method is to construct iterative transformations of the differential system (1) with quasi-periodic coefficients to the normal form (Birkhoff 1927). The system (1) is subjected to the normalizing iterative transformations excluding all short-period terms and leading to the secular system with slowly changing variables. As a result, one obtains the solution of the secular system avoiding the appearance of the non-physical secular terms.

The most cumbersome operation of GPT-method is the Poincaré normalization of the differential system (1). The evaluation problem in celestial mechanics is of particular importance owing to the large number of terms in the series. The analytical calculations in GPT-method are performed by the Poisson series processor (Brumberg 1995; Ivanova 2001).

In the present paper we develop a recursive algorithm based on Carleman linearization for computing the series. The algorithm provides explicit formulas for the coefficients of the Poincaré normal form and the normalizing transformation. Therefore, the Carleman linearization technique may be advantageous for constructing normal forms in a literal form.

This paper is organized as follows. In the next section, we describe our notations. In sections ‘The Weierstrass matrix’ and ‘The Carleman matrix’, we study two classes of infinite matrices, corresponding to nonlinear mappings and differential systems. In section ‘Transformations’, we study the transformations of infinite matrices. Section ‘Normal form of a Carleman matrix’ presents the recursive algorithm for constructing the Poincaré normalization. The proofs of the propositions are given in section ‘Proofs’. Finally, we give an example and discuss the results.

**Notations**

- ${\mathbb {R}}$—real number field,
- ${\mathbb {C}}$—complex number field,
- ${\mathbb {K}}$—either real or complex number field,
- ${\mathbb {Z}}$—the ring of integer numbers,
- ${\mathbb {N}}_0$—the set of integer non-negative numbers,
- ${\mathbb {N}}_0^{\nu }\mathop {=}\limits ^{\mathrm {def}}\left\{ \mathbf {n}=(n_1,\ldots ,n_{\nu })\mid n_{\alpha }\in {\mathbb {N}}_0\right\}$—the set of multi-indices,
- $$|{\mathbf {n}}|\mathop {=}\limits ^{\mathrm {def}}\sum \nolimits _{\alpha =1}^{\nu }n_{\alpha }$$,
- $${\mathbf {X}}^{\mathbf {n}}\mathop {=}\limits ^{\mathrm {def}}X_1^{n_1}\times \cdots \times X_{\nu }^{n_\nu }$$,
- $${\mathbf {e}}_{\alpha }\mathop {=}\limits ^{\mathrm {def}}(0,\ldots ,\underbrace{1}_{\alpha },\ldots ,0)$$,
- $\prec$—the precedence sign in ${\mathbb {N}}_0^{\nu }$,
- $\preceq$—the sign of precedence or equality in ${\mathbb {N}}_0^{\nu }$,
- $$({\mathbf {m}}:{\mathbf {n}})\mathop {=}\limits ^{\mathrm {def}}\left\{ \mathbf {l}\in {\mathbb {N}}_0^{\nu }\mid {\mathbf {m}} \prec {\mathbf {l}}\prec {\mathbf {n}}\right\}$$,
- $$({\mathbf {m}}:{\mathbf {n}}]\mathop {=}\limits ^{\mathrm {def}}\left\{ {\mathbf {l}}\in {\mathbb {N}}_0^{\nu }\mid {\mathbf {m}}\prec {\mathbf {l}}\preceq {\mathbf {n}}\right\}$$,
- $$[{\mathbf {m}}:{\mathbf {n}})\mathop {=}\limits ^{\mathrm {def}}\left\{ {\mathbf {l}}\in {\mathbb {N}}_0^{\nu }\mid {\mathbf {m}}\preceq {\mathbf {l}}\prec {\mathbf {n}}\right\}$$,
- $$I(\mu ,\nu )\mathop {=}\limits ^{\mathrm {def}}\left\{ {\mathbf {n}}\in {\mathbb {N}}_0^{\nu }\mid |{\mathbf {n}}|=\mu \right\}$$,
- $$s(\mu ,\nu )\mathop {=}\limits ^{\mathrm {def}}card(I(\mu ,\nu ))=\dfrac{(\mu +\nu -1)!}{(\nu -1)!\mu !}$$,
- $$\langle ,\rangle$$—scalar product,
- *A*—algebra over ${\mathbb {K}}$ of quasi-periodic functions ${\mathbb {R}}\rightarrow {\mathbb {K}}$, defined by ${\mathbb {K}}$-valued finite trigonometric sums,
- $\dfrac{d}{dt}$—differential operator in *A*.

## The Weierstrass matrix

Let $A[\nu ]$ be algebra over ${\mathbb {K}}$ of mappings ${\mathbb {R}}\times {\mathbb {K}}^{\nu }\rightarrow {\mathbb {K}}^{\nu }$, represented by a formal power series:

$$Y_{\alpha }=g_{\alpha }(t,{\mathbf {X}})=\sum \limits _{\mu =1}^{\infty }\sum \limits _{{\mathbf {n}}\in I(\mu ,\nu )}g[{\mathbf {e}}_{\alpha },{\mathbf {n}}]\,{\mathbf {X}}^{\mathbf {n}},$$

with $${\mathbf {X}},\,{\mathbf {Y}}\in {\mathbb {K}}^{\nu },\,g[{\mathbf {e}}_{\alpha },{\mathbf {n}}]\in A,\,\alpha =1,\ldots ,\nu$$.

We introduce the countable sets of variables:

2 $$\left\{ x[{\mathbf {n}}]={\mathbf {X}}^{\mathbf {n}}\mid {\mathbf {n}}\in {\mathbb {N}}_0^{\nu }\right\} ,\,\left\{ y[{\mathbf {n}}]={\mathbf {Y}}^{\mathbf {n}}\mid {\mathbf {n}}\in {\mathbb {N}}_0^{\nu }\right\} .$$

Let $x[{\mathbf {n}}]=1$ for ${\mathbf {n}}\notin {\mathbb {N}}_0^{\nu }$ and $g[{\mathbf {m}},{\mathbf {n}}]=0$ for $({\mathbf {m}}\notin {\mathbb {N}}_0^{\nu })\vee ({\mathbf {n}}\notin {\mathbb {N}}_0^{\nu })\vee ({\mathbf {n}}=0)\vee ({\mathbf {m}}=0)$.

### **Proposition 1**

*The variables*$y[{\mathbf {m}}]$*and*$x[{\mathbf {m}}]$*satisfy the linear equations*

$$y[{\mathbf {m}}]=\sum \limits _{\mu =1}^{\infty }\sum \limits _{{\mathbf {n}}\in I(\mu ,\nu )}g[{\mathbf {m}},{\mathbf {n}}]\,x[{\mathbf {n}}]$$

*where the coefficients*$g[{\mathbf {m}},{\mathbf {n}}]$*for*$|{\mathbf {m}}|>1$*may be obtained by the recursion formula*

3 $$g[{\mathbf {m}},{\mathbf {n}}]=\dfrac{1}{|{\mathbf {m}}|}\sum \limits _{\mu =|{\mathbf {m}}|-1}^{|{\mathbf {n}}|-1}\sum \limits _{{\mathbf {k}}\in I(\mu ,\nu )}\sum \limits _{\alpha =1}^{\nu }m_{\alpha }g[{\mathbf {m}}-{\mathbf {e}}_{\alpha },{\mathbf {k}}]\,g[{\mathbf {e}}_{\alpha },{\mathbf {n}}-{\mathbf {k}}].$$

We introduce natural ordering for the set ${\mathbb {N}}_0^{\nu }$. Let ${\mathbf {k}}\prec {\mathbf {l}}$ for ${\mathbf {k}},\,{\mathbf {l}}\in {\mathbb {N}}_0^{\nu }$, if $|{\mathbf {k}}|<|{\mathbf {l}}|$ or $|{\mathbf {k}}|=|{\mathbf {l}}|$ and there exists a number $\beta$ such that $k_{\alpha }=l_{\alpha }$ for $\alpha <\beta$ and $k_{\beta }>l_{\beta }$.

Using the ordering of ${\mathbb {N}}_0^{\nu }$, we introduce infinite-dimensional vectors ${\mathbf {x}}=(x[{\mathbf {n}}])$, ${\mathbf {y}}=(y[{\mathbf {n}}])$ and the matrix ${\mathbf {G}}=(g[{\mathbf {m}},{\mathbf {n}}])$ for ${\mathbf {m}},\,{\mathbf {n}}\in {\mathbb {N}}_0^{\nu }$ such that

$${\mathbf {y}}={\mathbf {G}}{\mathbf {x}}.$$

### **Definition 1**

The infinite matrix ${\mathbf {G}}$ is said to be a Weierstrass matrix with a range $\nu$

$${\mathbf {G}}\in W^{\nu }(A)$$

if the elements $g[{\mathbf {m}},{\mathbf {n}}]\in A$ satisfy the condition given by (3) for $|{\mathbf {m}}|>1$.

Let $\psi$ denote the constructed correspondence between mappings and matrices

$$\psi :A[\nu ]\rightarrow W^{\nu }(A).$$

The following proposition describes the structure of Weierstrass matrices.

### **Proposition 2**

1. *A Weierstrass matrix*${\mathbf {G}}$*consists of rectangular blocks*${\mathbf {G}}_{\alpha ,\beta }=(s(\alpha ,\nu )\times s(\beta ,\nu ))$*such that*${\mathbf {G}}_{\alpha ,\beta }=0$*for*$\alpha >\beta$.
2. *Let the Jacobi matrix*$\left. \left( \dfrac{\partial {\mathbf {g}}}{\partial {\mathbf {X}}}\right) \right| _{X=0}$*for*${\mathbf {g}}\in A[\nu ]$*be upper triangular with a main diagonal*${\varvec{\lambda }}=(\lambda _1,\ldots ,\lambda _{\nu })\in {\mathbb {K}}^{\nu }$. *Then*, $\psi ({\mathbf {g}})$*is an upper triangular matrix with main diagonal elements*$g[n,n]={\varvec{\lambda }}^{\mathbf {n}}$.

### **Definition 2**

1. The mapping ${\mathbf {g}}\in A[\nu ]$ is said to be invertible if there exists ${\mathbf {g}}^{-1}\in A[\nu ]$ such that ${\mathbf {g}}\circ {\mathbf {g}}^{-1}=\mathbf {1}_{A[\nu ]}$—identity mapping.
2. A matrix ${\mathbf {G}}\in W^{\nu }(A)$ is said to be invertible if there exists ${\mathbf {G}}^{-1}\in W^{\nu }(A)$ such that ${\mathbf {G}}\cdot {\mathbf {G}}^{-1}={\mathbf {E}}$.

### **Proposition 3**

*Weierstrass matrices*$W^{\nu }(A)$*form a semi-group with multiplication, which is isomorphic to the semi-group of mappings*$A[\nu ]$*with composition:*$\psi ({\mathbf {f}}\circ {\mathbf {g}})=\psi ({\mathbf {f}})\cdot \psi ({\mathbf {g}})$*for*${\mathbf {f}},\,{\mathbf {g}}\in A[\nu ]$.

### **Corollary 1**

*Invertible Weierstrass matrices*$W^{\nu }(A)$*form a group with multiplication, which is isomorphic to the group of invertible mappings*$A[\nu ]$*with composition.*

### **Corollary 2**

*Let*${\mathbf {f}},\,{\mathbf {g}}\in A[\nu ]$*and*${\mathbf {g}}$*be invertible. We introduce the mapping*$b_{\mathbf {g}}:{\mathbf {f}}\rightarrow {\mathbf {g}}^{-1}\circ {\mathbf {f}}\circ {\mathbf {g}}$. *Let*${\mathbf {F}},\,{\mathbf {G}}\in W^{\nu }(A)$*and*${\mathbf {G}}$*be invertible. We introduce the mapping*$B_{\mathbf {G}}:{\mathbf {F}}\rightarrow {\mathbf {G}}^{-1}{\mathbf {F}}{\mathbf {G}}$.

*Then, the following diagram is commutative*
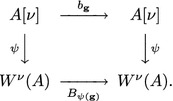


## The Carleman matrix

Here, we construct a class of infinite matrices representing ordinary differential equations.

Let $A[\nu ]$ denote linear space over ${\mathbb {K}}$ of vector fields ${\mathbf {f}}=(f_1,\ldots ,f_\nu )\in A[\nu ]$, represented by a formal power series:

4 $$\dfrac{d}{dt}\,{\mathbf {X}}={\mathbf {f}}({\mathbf {X}},t),\quad f_{\alpha }({\mathbf {X}},t)=\sum \limits _{\mu =1}^{\infty }\sum \limits _{{\mathbf {n}}\in I(\mu ,\nu )}f[{\mathbf {e}}_{\alpha },{\mathbf {n}}]\,{\mathbf {X}}^{\mathbf {n}}$$

for $${\mathbf {X}}\in {\mathbb {K}}^{\nu },\,t\in {\mathbb {R}},\,\alpha =1,\ldots ,\nu$$.

Let $f[{\mathbf {e}}_{\alpha },{\mathbf {n}}]=0$ for ${\mathbf {n}}\notin {\mathbb {N}}_0^{\nu }$ or ${\mathbf {n}}={\mathbf {0}}$.

### **Proposition 4**

*The variables*$x[{\mathbf {n}}]$ (2) *satisfy the following differential equations:*

$$\dfrac{d}{dt}\,x[{\mathbf {m}}]=\sum \limits _{\mu =|{\mathbf {m}}|}^{\infty }\sum \limits _{{\mathbf {n}}\in I(\mu ,\nu )}f[{\mathbf {m}},{\mathbf {n}}]\,x[{\mathbf {n}}],$$

*where the coefficients*$f[{\mathbf {m}},{\mathbf {n}}]$*for*$|{\mathbf {m}}|>1$*may be obtained from* (4) *by the formula*

5 $$f[{\mathbf {m}},{\mathbf {n}}]=\sum \limits _{\alpha =1}^{\nu }m_{\alpha }f[{\mathbf {e}}_{\alpha },{\mathbf {n}}-{\mathbf {m}}+{\mathbf {e}}_{\alpha }].$$

Using the ordering of ${\mathbb {N}}_0^{\nu }$, we introduce the infinite-dimensional vector ${\mathbf {x}}=(x[{\mathbf {n}}])$ and matrix ${\mathbf {F}}=(f[{\mathbf {m}},{\mathbf {n}}])$ for ${\mathbf {m}},\,{\mathbf {n}}\in {\mathbb {N}}_0^{\nu }$ such that:

6 $$\dfrac{d'}{dt}\,{\mathbf {x}}={\mathbf {F}}{\mathbf {x}},$$

where $\dfrac{d'}{dt}$ denotes differentiation of components.

### **Definition 3**

The infinite matrix ${\mathbf {F}}$ is said to be a Carleman matrix of range $\nu$

$${\mathbf {F}}\in C^{\nu }(A)$$

if the elements $f[{\mathbf {m}},{\mathbf {n}}]\in A$ for $|{\mathbf {m}}|>1$ satisfy the condition (5).

Let $\varphi$ denote the constructed correspondence between vector fields and matrices

$$\varphi :A[\nu ]\rightarrow C^{\nu }(A).$$

It is easy to see that $\varphi$ is an isomorphism of linear spaces. The following proposition describes the structure of the Carleman matrix.

### **Proposition 5**

1. *The Carleman matrix*${\mathbf {F}}$*consists of rectangular blocks*${\mathbf {F}}_{\alpha ,\beta }=(s(\alpha ,\nu )\times s(\beta ,\nu ))$*such that*${\mathbf {F}}_{\alpha ,\beta }=0$*for*$\alpha >\beta$.
2. *Let the Jacobi matrix*$\left. \left( \dfrac{\partial {\mathbf {f}}}{\partial {\mathbf {X}}}\right) \right| _{{\mathbf {X}}=0}$*for*${\mathbf {f}}\in A[\nu ]$*be upper triangular with a main diagonal*${\varvec{\lambda }}=(\lambda _1,\ldots ,\lambda _{\nu })\in {\mathbb {K}}^{\nu }$. *Then*, $\varphi ( {\mathbf {f}})$*is an upper triangular matrix with main diagonal elements*$f[{\mathbf {n}},{\mathbf {n}}]=\langle {\mathbf {n}},{\varvec{\lambda }}\rangle$.

## Transformations

Now, we consider the substitution

7 $${\mathbf {x}}={\mathbf {G}}{\mathbf {y}}$$

defined by the Weierstrass matrix ${\mathbf {G}}$ into differential equation (6) defined by the Carleman matrix ${\mathbf {F}}$. We prove that the result of the substitution (7) gives the differential equation defined by the Carleman matrix (Proposition 6) and may be interpreted in terms of vector fields (Proposition 7).

### **Proposition 6**

*Let*${\mathbf {G}}\in W^{\nu }(A)$*be invertible*, ${\mathbf {F}}\in C^{\nu }(A)$. *Let*$A_{\mathbf {G}}$*denote the mapping*

$$A_{\mathbf {G}}({\mathbf {F}})={\mathbf {G}}^{-1}\left( {\mathbf {F}}{\mathbf {G}}-\dfrac{d'}{dt}\,{\mathbf {G}}\right) .$$

*Then*, $$A_{\mathbf {G}}:C^{\nu }(A)\rightarrow C^{\nu }(A)$$.

### **Proposition 7**

*Let*${\mathbf {g}}(t,{\mathbf {X}})\in A[\nu ]$*be invertible and*${\mathbf {J}}_{\mathbf {g}}=\left( \dfrac{\partial {\mathbf {g}}}{\partial {\mathbf {X}}}\right)$. *Let*$a_{\mathbf {g}}:A[\nu ]\rightarrow A[\nu ]$*denote the mapping*

$$a_{\mathbf {g}}({\mathbf {f}})={\mathbf {J}}_{\mathbf {g}}^{-1}\left( {\mathbf {f}}\circ {\mathbf {g}}-\dfrac{\partial {\mathbf {g}}}{\partial t}\right) .$$

*Then, the following diagram is commutative:*
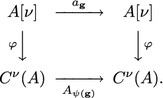


## Normal form of a Carleman matrix

In this section, we provide a definition of the normal form of a Carleman matrix. We also introduce a method for reducing the corresponding differential equations to the normal form.

Let $C_T^{\nu }(A)$ denote the linear space over ${\mathbb {K}}$ of upper triangular Carleman matrices with main diagonal elements from ${\mathbb {K}}$. Let $W_T^{\nu }(A)$ denote the semi-group of upper triangular Weierstrass matrices with main diagonal elements from ${\mathbb {K}}$.

Let *A* be the algebra over ${\mathbb {K}}$ of ${\mathbb {K}}$-valued finite trigonometric sums

$$\sum \limits _{{\mathbf {k}}\in {\mathbb {Z}}^{\sigma }}a[{\mathbf {k}}]\,exp\langle i {\varvec{\omega }} t,{\mathbf {k}}\rangle \quad {\text{ for }}\ a[{\mathbf {k}}]\in {\mathbb {C}},\quad t\in {\mathbb {R}}$$

which determine quasi-periodic functions of *t* with a frequency set ${\varvec{\omega }}\in {\mathbb {R}}^{\sigma }$. If ${\mathbb {K}}={\mathbb {R}}$, let $a[k]=\overline{a[-k]}$.

Consider the matrix ${{\mathbf {H}}}\in C_T^{\nu }(A)$ with elements

$$h[{\mathbf {m}},{\mathbf {n}}]=\sum \limits _{{\mathbf {k}}\in {\mathbb {Z}}^{\sigma }}h[{\mathbf {m}},{\mathbf {n}},{\mathbf {k}}]\,exp\langle i {\varvec{\omega }} t,{\mathbf {k}}\rangle .$$

Let ${\varvec{\lambda }}\in {\mathbb {K}}^{\nu }$ denote the first $\nu$ main diagonal elements of ${{\mathbf {H}}}:\lambda _{\alpha }=h[{\mathbf {e}}_{\alpha },{\mathbf {e}}_{\alpha }]\,$, $\alpha =1,\ldots ,\nu$.

### **Definition 4**

$({\mathbf {m}},{\mathbf {n}},{\mathbf {k}})$—resonance holds if the following condition is satisfied:

$$\langle {\mathbf {m}},{\varvec{\lambda }}\rangle =\langle {\mathbf {n}},{\varvec{\lambda }}\rangle +i\langle {\mathbf {k}},{\varvec{\omega }}\rangle ,$$

where ${\mathbf {m}},\,{\mathbf {n}}\in {\mathbb {N}}_0^{\nu },\, {\mathbf {k}}\in {\mathbb {Z}}^{\sigma }$. The harmonic $h[{\mathbf {m}},{\mathbf {n}},{\mathbf {k}}]\,exp\langle i{\varvec{\omega }} t,k\rangle$ is said to be resonant if $({\mathbf {m}},{\mathbf {n}},{\mathbf {k}})$—resonance holds. A Carleman matrix ${{\mathbf {H}}}$ is reduced to the normal form if all of its non-zero harmonics are resonant.

### **Theorem**

*For any Carleman matrix*${\mathbf {F}}\in C_T^{\nu }(A)$, *there exists an invertible Weierstrass matrix*${\mathbf {G}}\in W_T^{\nu }(A)$*reducing*${\mathbf {F}}$*to the normal form*${{\mathbf {H}}}\in C_T^{\nu }(A)$:

$${{\mathbf {H}}}=A_{\mathbf {G}}({\mathbf {F}}).$$

### Proof

We obtain the components of ${\mathbf {G}}$ and ${{\mathbf {H}}}$ in the following order:

8 $$(1,1)\rightarrow (2,2)\rightarrow (1,2)\rightarrow (3,3)\rightarrow (2,3)\rightarrow (1,3)\rightarrow \cdots .$$

We restrict ourselves to the case $g[{\mathbf {n}},{\mathbf {n}}]=1$ for ${\mathbf {n}}\in {\mathbb {N}}_0^{\nu }$.

It follows from the equation ${\mathbf {F}}{\mathbf {G}}-\dfrac{d'}{dt}\,{\mathbf {G}}={\mathbf {G}}{{\mathbf {H}}}$ that we can make the non-resonant harmonic $h[{\mathbf {e}}_{\alpha },{\mathbf {n}},{\mathbf {k}}]exp\langle i{\varvec{\omega }} t,{\mathbf {k}}\rangle$ vanish by a proper choice of each $g[{\mathbf {e}}_{\alpha },{\mathbf {n}},{\mathbf {k}}]$, namely

9 $$\begin{aligned} g[{\mathbf {e}}_{\alpha },{\mathbf {n}},{\mathbf {k}}]&= \dfrac{1}{\langle {\mathbf {n}}-{\mathbf {e}}_{\alpha }, {\varvec{\lambda }}\rangle +i\langle {\varvec{\omega }},{\mathbf {k}}\rangle }\times \left( \sum \limits _{{\mathbf {i}}\in ({\mathbf {e}}_{\alpha }:{\mathbf {n}}]}\sum \limits _{\mathbf {a}\in {\mathbb {Z}}^{\sigma }}f[{\mathbf {e}}_{\alpha }, {{\mathbf {i}}}, {\mathbf {a}}]\,g[{{\mathbf {i}}},{\mathbf {n}},{\mathbf {k}} -{\mathbf {a}}]\right. \\&\quad \left. -\sum \limits _{{\mathbf {j}}\in [{\mathbf {e}}_{\alpha }: {\mathbf {n}})}\sum \limits _{\mathbf {b}\in {\mathbb {Z}}^{\sigma }}g[{\mathbf {e}}_{\alpha }, {\mathbf {j}},{\mathbf {b}}]\,h[{\mathbf {j}},{\mathbf {n}},{\mathbf {k}}- {\mathbf {b}}]\right) . \end{aligned}$$

If $({\mathbf {e}}_{\alpha },{\mathbf {n}},{\mathbf {k}})$—resonance holds, we cannot eliminate the corresponding resonant harmonic via a choice of $g[{\mathbf {e}}_{\alpha },{\mathbf {n}},{\mathbf {k}}]$. In this case, $g[{\mathbf {e}}_{\alpha },{\mathbf {n}},{\mathbf {k}}]$ may be assigned an arbitrary value. Then, one obtains the resonant harmonic in ${\mathbf {H}}$ as follows

10 $$\begin{aligned} h[{\mathbf {e}}_{\alpha },{\mathbf {n}},{\mathbf {k}}]&=\sum \limits _{{\mathbf {i}}\in [{\mathbf {e}}_{\alpha }:{\mathbf {n}}]} \sum \limits _{\mathbf {a}\in {\mathbb {Z}}^{\sigma }}f[{\mathbf {e}}_{\alpha },{{\mathbf {i}}}, {\mathbf {a}}]\,g[{{\mathbf {i}}},{\mathbf {n}},{\mathbf {k}}-{\mathbf {a}}] \\&\quad -\sum \limits _{{\mathbf {j}}\in ({\mathbf {e}}_{\alpha }:{\mathbf {n}}]}\sum \limits _{\mathbf {b}\in {\mathbb {Z}}^{\sigma }}g[{\mathbf {e}}_{\alpha }, {\mathbf {j}},{\mathbf {b}}]\,h[{\mathbf {j}},{\mathbf {n}},{\mathbf {k}} -{\mathbf {b}}]-\langle i{\varvec{\omega }},{\mathbf {k}}\rangle \,g[{\mathbf {e}}_{\alpha }, {\mathbf {n}},{\mathbf {k}}]. \end{aligned}$$

By Proposition 6, one obtains the components of ${\mathbf {G}}$ and ${\mathbf {H}}$ below the $\nu$-row by (3) and (5), respectively. $\square$

### Remark 1

The leading $\nu$ rows of ${\mathbf {G}}$ and ${{\mathbf {H}}}$ determine the normalizing transformation ${\mathbf {X}}=g(t,{\mathbf {Y}})$ and the normal form of the differential equation $\dfrac{d}{dt}{\mathbf {X}}={\mathbf {h}}(t,{\mathbf {X}})$, respectively, where ${\mathbf {G}}=\psi ({\mathbf {g}})$ and ${{\mathbf {H}}}=\varphi ({\mathbf {h}})$.

### Remark 2

The elements of the inverse matrix ${\mathbf {G}}^{-1}=(g^*[{\mathbf {m}},{\mathbf {n}}])$ may be obtained together with the elements of ${\mathbf {G}}$ in the order (8). By equation ${\mathbf {G}}^{-1}{\mathbf {G}}={\mathbf {E}}$, one obtains that:

$$g^*[{\mathbf {m}},{\mathbf {n}}]=-\sum \limits _{{\mathbf {k}} \in [{\mathbf {m}}:{\mathbf {n}})}g^*[{\mathbf {m}},{\mathbf {k}}]\,g[{\mathbf {k}}, {\mathbf {n}}].$$

## Proofs

In this section, we provide proofs of the propositions introduced above.

### Proof of Proposition 1

Using the properties of homogeneous polynomials, we obtain:

$$\begin{aligned} y[{\mathbf {m}}]&= {{\mathbf {Y}}}^{\mathbf {m}}=\sum \limits _{\alpha =1}^{\nu } \dfrac{1}{|{\mathbf {m}}|}\dfrac{\partial {{\mathbf {Y}}}^m}{\partial Y_{\alpha }}\,Y_{\alpha }=\dfrac{1}{|{\mathbf {m}}|} \sum \limits _{\alpha =1}^{\nu }m_{\alpha }y[{\mathbf {m}}-{\mathbf {e}}_{\alpha }] \,y[{\mathbf {e}}_{\alpha }]\\&= \dfrac{1}{|{\mathbf {m}}|}\sum \limits _{\gamma =|{\mathbf {m}}|-1}^{\infty }\sum \limits _{{\mathbf {k}}\in I(\gamma ,\nu )}\sum \limits _{\delta =1}^{\infty }\sum \limits _{{\mathbf {l}}\in I(\delta ,\nu )}\left( \sum \limits _{\alpha =1}^{\nu }m_{\alpha }g[{\mathbf {m}} -{\mathbf {e}}_{\alpha },{\mathbf {k}}]\,g[{\mathbf {e}}_{\alpha },{\mathbf {l}}]\right) x[{\mathbf {k}}+{\mathbf {l}}]\\&= \dfrac{1}{|{\mathbf {m}}|}\sum \limits _{\gamma =|{\mathbf {m}}|}^{\infty }\sum \limits _{{\mathbf {n}}\in I(\gamma ,\nu )}\sum \limits _{\mu =|{\mathbf {m}}|-1}^{\gamma -1}\sum \limits _{{\mathbf {k}}\in I(\mu ,\nu )}\left( \sum \limits _{\alpha =1}^{\nu }m_{\alpha } g[{\mathbf {m}}-{\mathbf {e}}_{\alpha },{\mathbf {k}}]\,g[{\mathbf {e}}_{\alpha },{\mathbf {n}} -{\mathbf {k}}]\right) x[{\mathbf {n}}]. \end{aligned}$$

$$\square$$

### Proof of Proposition 2

Part 1. It is an immediate consequence of the definition provided in (3).

Part 2. Let ${\mathbf {G}}=\psi (g)$. We prove that $g[{\mathbf {m}},{\mathbf {n}}]\ne 0$ for ${\mathbf {m}}\preceq {\mathbf {n}}$ only. By (3), $g[{\mathbf {m}},{\mathbf {n}}]\ne 0$ yields

$$\left( ({\mathbf {m}}-{\mathbf {e}}_{\alpha })\in {\mathbb {N}}_0^{\nu }\right) \wedge \left( ({\mathbf {n}}-{\mathbf {k}})\in {\mathbb {N}}_0^{\nu }\right) \wedge \left( ({\mathbf {m}}-{\mathbf {e}}_{\alpha })\preceq {\mathbf {k}}\right) \wedge \left( {\mathbf {e}}_{\alpha }\preceq ({\mathbf {n}}-{\mathbf {k}})\right)$$

for at least one $\alpha =1,\ldots ,\nu$ and one ${\mathbf {k}}\in \bigcup \nolimits _{\mu =|{\mathbf {m}}|-1}^{|{\mathbf {n}}|-1}I(\mu ,\nu )$. It accordingly follows that ${\mathbf {m}}\preceq {\mathbf {n}}$. $\square$

For later considerations, it will be useful to prove a property of Weierstrass matrices.

### **Lemma 1**

*Let*${\mathbf {G}}=(g[{\mathbf {m}},{\mathbf {n}}])\in W^{\nu }(A)$*and*${{\mathbf {i}}},\,{\mathbf {j}},\,{\mathbf {n}}\in {\mathbb {N}}_0^{\nu }$. *Then:*

$$g[{{\mathbf {i}}}+ {\mathbf {j}},{\mathbf {n}}]=\sum \limits _{\mu =| {{\mathbf {i}}}|}^{|{\mathbf {n}}|-| {\mathbf {j}}|}\sum \limits _{{\mathbf {k}}\in I(\mu ,\nu )}g[{{\mathbf {i}}},{\mathbf {k}}]\,g[ {\mathbf {j}},{\mathbf {n}}-{\mathbf {k}}].$$

### Proof

$$\begin{aligned} y[{{\mathbf {i}}}+{\mathbf {j}}] &=y[{{\mathbf {i}}}]\,y[{\mathbf {j}}] =\sum \limits _{\mu =|{{\mathbf {i}}}|}^{\infty }\sum \limits _{{\mathbf {k}}\in I(\mu ,\nu )}g[{{\mathbf {i}}}, {\mathbf {k}}]\,x[{\mathbf {k}}]\sum \limits _{\delta =| {\mathbf {j}}|}^{\infty }\sum \limits _{{\mathbf {l}}\in I(\delta ,\nu )}g[{\mathbf {j}},{\mathbf {l}}]\,x[{\mathbf {l}}]\\&=\sum \limits _{\gamma =|{{\mathbf {i}}}|+|{\mathbf {j}}|}^{\infty }\sum \limits _{{\mathbf {n}}\in I(\gamma ,\nu )}\left( \sum \limits _{\mu =|{\mathbf {i}}|}^{|{\mathbf {n}}|-|{\mathbf {j}}|}\sum \limits _{{\mathbf {k}}\in I(\mu ,\nu )}g[{\mathbf {i}},{\mathbf {k}}]\,g[{\mathbf {j}}, {\mathbf {n}}-{\mathbf {k}}]\right) x[{\mathbf {n}}]. \end{aligned}$$

$$\square$$

### Remark 3

Another definition of a Weierstrass matrix may be introduced. Let $\alpha =1,\ldots ,\nu$ and ${\mathbf {m}}\in {\mathbb {N}}_0^{\nu }$. Then:

11 $$g[{\mathbf {m}}+{\mathbf {e}}_{\alpha },{\mathbf {n}}]=\sum \limits _{\mu =1}^{|{\mathbf {n}}|-|{\mathbf {m}}|}\sum \limits _{{\mathbf {k}}\in I(\mu ,\nu )}g[{\mathbf {e}}_{\alpha },{\mathbf {k}}]\,g[{\mathbf {m}},{\mathbf {n}}-{\mathbf {k}}].$$

### Proof of Proposition 3

Let ${\mathbf {F}},\,{\mathbf {G}}\in W^{\nu }(A)$ and ${\mathbf {H}}={\mathbf {F}}{\mathbf {G}}$. We prove that ${\mathbf {H}}\in W^{\nu }(A)$. We have

$$\begin{aligned} h[{\mathbf {m}}+{\mathbf {e}}_{\alpha },{\mathbf {n}}]&= \sum \limits _{\gamma =|{\mathbf {m}}|+1}^{|{\mathbf {n}}|}\sum \limits _{{\mathbf {i}}\in I(\gamma ,\nu )}f[{\mathbf {m}}+{\mathbf {e}}_{\alpha },{\mathbf {i}}]\,g[{\mathbf {i}},{\mathbf {n}}]\\&= \sum \limits _{\gamma =|{\mathbf {m}}|}^{|{\mathbf {n}}|}\sum \limits _{{\mathbf {i}}\in I(\gamma ,\nu )}g[{\mathbf {i}},{\mathbf {n}}]\sum \limits _{\delta =1}^{\gamma -|{\mathbf {m}}|}\sum \limits _{{\mathbf {j}}\in I(\delta ,\nu )}f[{\mathbf {e}}_{\alpha },{\mathbf {j}}]\,f[{\mathbf {m}},{\mathbf {i}}-{\mathbf {j}}]\\&= \sum \limits _{\delta =1}^{|{\mathbf {n}}|-|{\mathbf {m}}|}\sum \limits _{{\mathbf {j}}\in I(\delta ,\nu )}f[{\mathbf {e}}_{\alpha },{\mathbf {j}}]\sum \limits _{\gamma =|{\mathbf {m}}|}^{|{\mathbf {n}}|}\sum \limits _{{\mathbf {i}}\in I(\gamma ,\nu )}g[{\mathbf {i}},{\mathbf {n}}]\,f[{\mathbf {m}},{\mathbf {i}}-{\mathbf {j}}]\\&= \sum \limits _{\delta =1}^{|{\mathbf {n}}|-|{\mathbf {m}}|}\sum \limits _{{\mathbf {j}}\in I(\delta ,\nu )}f[{\mathbf {e}}_{\alpha },{\mathbf {j}}]\sum \limits _{\gamma =|{\mathbf {m}}|}^{|{\mathbf {n}}|}\sum \limits _{{\mathbf {l}}\in I(\gamma ,\nu )}g[{\mathbf {l}}+{\mathbf {j}},{\mathbf {n}}]\,f[{\mathbf {m}},{\mathbf {l}}]\\&= \sum \limits _{\delta =1}^{|{\mathbf {n}}|-|{\mathbf {m}}|}\sum \limits _{{\mathbf {j}}\in I(\delta ,\nu )}f[{\mathbf {e}}_{\alpha },{\mathbf {j}}]\sum \limits _{\gamma =|{\mathbf {m}}|}^{|{\mathbf {n}}|}\sum \limits _{{\mathbf {l}}\in I(\gamma ,\nu )}f[{\mathbf {m}},{\mathbf {l}}]\sum \limits _{\varepsilon =\delta }^{|{\mathbf {n}}|-\gamma }\sum \limits _{{\mathbf {k}}\in I(\varepsilon ,\nu )}g[{\mathbf {j}},{\mathbf {k}}]\,g[{\mathbf {l}},{\mathbf {n}}-{\mathbf {k}}]\\&= \sum \limits _{\varepsilon =1}^{|{\mathbf {n}}|-|{\mathbf {m}}|}\sum \limits _{{\mathbf {k}}\in I(\varepsilon ,\nu )}\left( \sum \limits _{\delta =1}^{|{\mathbf {n}}|-|{\mathbf {m}}|}\sum \limits _{{\mathbf {j}}\in I(\delta ,\nu )}f[{\mathbf {e}}_{\alpha },{\mathbf {j}}]\,g[{\mathbf {j}},{\mathbf {k}}]\right) \\&\quad \times \left( \sum \limits _{\gamma =|{\mathbf {m}}|}^{|{\mathbf {n}}|}\sum \limits _{{\mathbf {l}}\in I(\gamma ,\nu )}f[{\mathbf {m}},{\mathbf {l}}]\,g[{\mathbf {l}},{\mathbf {n}}-{\mathbf {k}}]\right) \\&= \sum \limits _{\varepsilon =1}^{|{\mathbf {n}}|-|{\mathbf {m}}|}\sum \limits _{{\mathbf {k}}\in I(\varepsilon ,\nu )}h[{\mathbf {e}}_{\alpha },{\mathbf {k}}]\,h[{\mathbf {m}},{\mathbf {n}}-{\mathbf {k}}]. \end{aligned}$$

Condition (11) is satisfied, i.e., ${{\mathbf {H}}}\in W^{\nu }(A)$.  $\square$

### Proof of Proposition 4

One obtains that:

$$\begin{aligned} \dfrac{d}{dt}\,x[{\mathbf {m}}]&= \sum \limits _{\alpha =1}^{\nu }m_{\alpha }x[{\mathbf {m}}-{\mathbf {e}}_{\alpha }]\,\dfrac{d}{dt}\,x[{\mathbf {e}}_{\alpha }]\\&= \sum \limits _{\mu =1}^{\infty }\sum \limits _{{\mathbf {k}}\in I(\mu ,\nu )}\sum \limits _{\alpha =1}^{\nu }m_{\alpha }f[{\mathbf {e}}_{\alpha },{\mathbf {k}}]\,x[{\mathbf {k}}+{\mathbf {m}}-{\mathbf {e}}_{\alpha }]\\&= \sum \limits _{\mu =|{\mathbf {m}}|}^{\infty }\sum \limits _{{\mathbf {n}}\in I(\mu ,\nu )}\left( \sum \limits _{\alpha =1}^{\nu }m_{\alpha }f[{\mathbf {e}}_{\alpha },{\mathbf {n}}-{\mathbf {m}}+{\mathbf {e}}_{\alpha }]\right) x[{\mathbf {n}}]. \end{aligned}$$

This mathematics proves the proposition. $\square$

### Proof of Proposition 5

Part 1. It is an immediate consequence of definition (5) Part 2. We prove that $f[{\mathbf {m}},{\mathbf {n}}]\ne 0$ for ${\mathbf {m}}\preceq {\mathbf {n}}$ only. By (5), $f[{\mathbf {m}},{\mathbf {n}}]\ne 0$ yields:

$$\left( ({\mathbf {n}}-{\mathbf {m}}+{\mathbf {e}}_{\alpha })\in {\mathbb {N}}_0^{\nu }\right) \wedge \left( ({\mathbf {n}}-{\mathbf {m}}+{\mathbf {e}}_{\alpha })\succeq {\mathbf {e}}_{\alpha }\right)$$

for at least one $\alpha =1,\ldots ,\nu$. From this statement, it follows that ${\mathbf {m}}\preceq {\mathbf {n}}$.  $\square$

### **Lemma 2**

*Let*${\mathbf {F}}=(f[{\mathbf {n}},{\mathbf {m}}])\in C^{\nu }(A)$*and*${\mathbf {i}},\,{\mathbf {j}},\,{\mathbf {n}}\in {\mathbb {N}}_0^{\nu }$. *Then:*

$$f[{\mathbf {i}}+{\mathbf {j}},{\mathbf {n}}]=f[{\mathbf {i}},{\mathbf {n}}-{\mathbf {j}}]+f[{\mathbf {j}},{\mathbf {n}}-{\mathbf {i}}].$$

### Proof

One obtains that:

$$\begin{aligned} \dfrac{d}{dt}\,x[{\mathbf {i}}+{\mathbf {j}}]&= x[{\mathbf {i}}]\,\dfrac{d}{dt}\,x[{\mathbf {j}}]+x[{\mathbf {j}}]\,\dfrac{d}{dt}\,x[{\mathbf {i}}]\\&= \sum \limits _{\varepsilon =|{\mathbf {j}}|}^{\infty }\sum \limits _{{\mathbf {q}}\in I(\varepsilon ,\nu )}f[{\mathbf {j}},{\mathbf {q}}]\,x[{\mathbf {q}}+{\mathbf {i}}]+\sum \limits _{\delta =|{\mathbf {i}}|}^{\infty }\sum \limits _{{\mathbf {p}}\in I(\delta ,\nu )}f[{\mathbf {i}},{\mathbf {p}}]\,x[{\mathbf {p}}+{\mathbf {j}}]\\&= \sum \limits _{\mu =|{\mathbf {i}}|+|{\mathbf {j}}|}^{\infty }\sum \limits _{{\mathbf {n}}\in I(\mu ,\nu )}\left( f[{\mathbf {i}},{\mathbf {n}}-{\mathbf {j}}]+f[{\mathbf {j}},{\mathbf {n}}-{\mathbf {i}}]\right) \,x[{\mathbf {n}}]. \end{aligned}$$

$$\square$$

### Remark 4

Another definition for the Carleman matrix may be introduced. Let $\alpha =1,\ldots ,\nu$ and ${\mathbf {m}}\in {\mathbb {N}}_0^{\nu }$. Then:

12 $$f[{\mathbf {m}}+{\mathbf {e}}_{\alpha },{\mathbf {n}}]=f[{\mathbf {m}},{\mathbf {n}}-{\mathbf {e}}_{\alpha }]+f[{\mathbf {e}}_{\alpha },{\mathbf {n}}-{\mathbf {m}}].$$

### Proof of Proposition 6

Step 1. Let ${\mathbf {H}}={\mathbf {G}}^{-1}{\mathbf {F}}{\mathbf {G}}$ and ${\mathbf {G}}^{-1}=(g^*[{\mathbf {m}},{\mathbf {n}}])$. We prove that ${\mathbf {H}}\in C^{\nu }(A)$. By (11), one obtains that:

$$\begin{aligned} h[{\mathbf {m}}+{\mathbf {e}}_{\alpha },{\mathbf {n}}]&= \sum \limits _{\gamma =|{\mathbf {m}}|+1}^{|{\mathbf {n}}|}\sum \limits _{{\mathbf {k}}\in I(\gamma ,\nu )}g^*[{\mathbf {m}}+{\mathbf {e}}_{\alpha },{\mathbf {k}}]\sum \limits _{\delta =\gamma }^{|{\mathbf {n}}|}\sum \limits _{{\mathbf {l}}\in I(\delta ,\nu )}f[{\mathbf {k}},{\mathbf {l}}]\,g[{\mathbf {l}},{\mathbf {n}}]\\&= \sum \limits _{\gamma =|{\mathbf {m}}|+1}^{|{\mathbf {n}}|}\sum \limits _{{\mathbf {k}}\in I(\gamma ,\nu )} \sum \limits _{\varepsilon =1}^{\gamma -|{\mathbf {m}}|}\sum \limits _{{\mathbf {p}}\in I(\varepsilon ,\nu )}g^*[{\mathbf {e}}_{\alpha },{\mathbf {p}}]g^*[{\mathbf {m}},{\mathbf {k}}-{\mathbf {p}}]\sum \limits _{\delta =\gamma }^{|{\mathbf {n}}|}\sum \limits _{{\mathbf {l}}\in I(\delta ,\nu )}f[{\mathbf {k}},{\mathbf {l}}]\,g[{\mathbf {l}},{\mathbf {n}}]\\&= \sum \limits _{\varepsilon =1}^{|{\mathbf {n}}|-|{\mathbf {m}}|}\sum \limits _{{\mathbf {p}}\in I(\varepsilon ,\nu )}g^*[{\mathbf {e}}_\alpha ,{\mathbf {p}}]\sum \limits _{\gamma =|{\mathbf {m}}|}^{|{\mathbf {n}}|-1}\sum \limits _{{\mathbf {i}}\in I(\gamma ,\nu )}g^*[{\mathbf {m}},{\mathbf {i}}]\sum \limits _{\delta =\varepsilon +\gamma }^{|{\mathbf {n}}|}\sum \limits _{{\mathbf {l}}\in I(\delta ,\nu )}f[{\mathbf {p}}+{\mathbf {i}},{\mathbf {l}}]\,g[{\mathbf {l}},{\mathbf {n}}]\\&= \Sigma _1+\Sigma _2, \end{aligned}$$

where

$$\begin{aligned} \Sigma _1&= \sum \limits _{\varepsilon =1}^{|{\mathbf {n}}|-|{\mathbf {m}}|}\sum \limits _{{\mathbf {p}}\in I(\varepsilon ,\nu )}g^*[{\mathbf {e}}_{\alpha },{\mathbf {p}}]\sum \limits _{\gamma =|{\mathbf {m}}|}^{|{\mathbf {n}}|-1}\sum \limits _{{\mathbf {i}}\in I(\gamma ,\nu )}g^*[{\mathbf {m}},{\mathbf {i}}]\sum \limits _{\delta =\varepsilon +\gamma }^{|{\mathbf {n}}|}\sum \limits _{{\mathbf {l}}\in I(\delta ,\nu )}f[{\mathbf {p}},{\mathbf {l}}-{\mathbf {i}}]\,g[{\mathbf {l}},{\mathbf {n}}]\\&= \sum \limits _{\varepsilon =1}^{|{\mathbf {n}}|-|{\mathbf {m}}|}\sum \limits _{{\mathbf {p}}\in I(\varepsilon ,\nu )}g^*[{\mathbf {e}}_{\alpha },{\mathbf {p}}]\sum \limits _{\gamma =|{\mathbf {m}}|}^{|{\mathbf {n}}|-1}\sum \limits _{{\mathbf {i}}\in I(\gamma ,\nu )}g^*[{\mathbf {m}},{\mathbf {i}}]\sum \limits _{\delta =\varepsilon }^{|{\mathbf {n}}|-\gamma }\sum \limits _{{\mathbf {r}}\in I(\delta ,\nu )}f[{\mathbf {p}},{\mathbf {r}}]\,g[{\mathbf {r}}+{\mathbf {i}},{\mathbf {n}}]\\&= \sum \limits _{\varepsilon =1}^{|{\mathbf {n}}|-|{\mathbf {m}}|}\sum \limits _{{\mathbf {p}}\in I(\varepsilon ,\nu )}g^*[{\mathbf {e}}_{\alpha },{\mathbf {p}}]\sum \limits _{\delta =\varepsilon }^{|{\mathbf {n}}|-|{\mathbf {m}}|}\sum \limits _{{\mathbf {r}}\in I(\delta ,\nu )}f[{\mathbf {p}},{\mathbf {r}}]\sum \limits _{\gamma =|{\mathbf {m}}|}^{|{\mathbf {n}}|-1}\sum \limits _{{\mathbf {i}}\in I(\gamma ,\nu )}g^*[{\mathbf {m}},{\mathbf {i}}]\,g[{\mathbf {r}}+{\mathbf {i}},{\mathbf {n}}]\\&= \sum \limits _{\varepsilon =1}^{|{\mathbf {n}}|-|{\mathbf {m}}|}\sum \limits _{{\mathbf {p}}\in I(\varepsilon ,\nu )}g^*[{\mathbf {e}}_{\alpha },{\mathbf {p}}]\sum \limits _{\delta =\varepsilon }^{|{\mathbf {n}}|-|{\mathbf {m}}|}\sum \limits _{{\mathbf {r}}\in I(\delta ,\nu )}f[{\mathbf {p}},{\mathbf {r}}]\sum \limits _{\gamma =|{\mathbf {m}}|}^{|{\mathbf {n}}|-1}\sum \limits _{{\mathbf {i}}\in I(\gamma ,\nu )}g^*[{\mathbf {m}},{\mathbf {i}}]\\&\quad \times \sum \limits _{\sigma =\gamma }^{|{\mathbf {n}}|-\delta }\sum \limits _{{\mathbf {s}}\in I(\sigma ,\nu )}g[{\mathbf {i}},{\mathbf {s}}]\,g[{\mathbf {r}},{\mathbf {n}}-{\mathbf {s}}]=\sum \limits _{\varepsilon =1}^{|{\mathbf {n}}|-|{\mathbf {m}}|}\sum \limits _{{\mathbf {p}}\in I(\varepsilon ,\nu )}g^*[{\mathbf {e}}_{\alpha },{\mathbf {p}}]\sum \limits _{\delta =\varepsilon }^{|{\mathbf {n}}|-|{\mathbf {m}}|}\sum \limits _{{\mathbf {r}}\in I(\delta ,\nu )}f[{\mathbf {p}},{\mathbf {r}}]\\&\quad \times \sum \limits _{\sigma =|{\mathbf {m}}|}^{|{\mathbf {n}}|-\delta }\sum \limits _{{\mathbf {s}}\in I(\sigma ,\nu )}g[{\mathbf {r}},{\mathbf {n}}-{\mathbf {s}}]\sum \limits _{\gamma =|{\mathbf {m}}|}^{|{\mathbf {n}}|-1}\sum \limits _{{\mathbf {i}}\in I(\gamma ,\nu )}g^*[{\mathbf {m}},{\mathbf {i}}]\,g[{\mathbf {i}},{\mathbf {s}}]\\&= \sum \limits _{\varepsilon =1}^{|{\mathbf {n}}|-|{\mathbf {m}}|}\sum \limits _{{\mathbf {p}}\in I(\varepsilon ,\nu )}g^*[{\mathbf {e}}_{\alpha },{\mathbf {p}}]\sum \limits _{\delta =\varepsilon }^{|{\mathbf {n}}|-|{\mathbf {m}}|}\sum \limits _{{\mathbf {r}}\in I(\delta ,\nu )}f[{\mathbf {p}},{\mathbf {r}}]\,g[{\mathbf {r}},{\mathbf {n}}-{\mathbf {m}}]=h[{\mathbf {e}}_{\alpha },{\mathbf {n}}-{\mathbf {m}}],\\ \Sigma _2&= \sum \limits _{\varepsilon =1}^{|{\mathbf {n}}|-|{\mathbf {m}}|}\sum \limits _{{\mathbf {p}}\in I(\varepsilon ,\nu )}g^*[{\mathbf {e}}_{\alpha },{\mathbf {p}}]\sum \limits _{\gamma =|{\mathbf {m}}|}^{|{\mathbf {n}}|-1}\sum \limits _{{\mathbf {i}}\in I(\gamma ,\nu )}g^*[{\mathbf {m}},{\mathbf {i}}]\sum \limits _{\delta =\varepsilon +\gamma }^{|{\mathbf {n}}|}\sum \limits _{{\mathbf {l}}\in I(\delta ,\nu )}f[{\mathbf {i}},{\mathbf {l}}-{\mathbf {p}}]\,g[{\mathbf {l}},{\mathbf {n}}]\\&= \sum \limits _{\gamma =|{\mathbf {m}}|}^{|{\mathbf {n}}|-1}\sum \limits _{{\mathbf {i}}\in I(\gamma ,\nu )}g^*[{\mathbf {m}},{\mathbf {i}}]\sum \limits _{\delta =|{\mathbf {m}}|}^{|{\mathbf {n}}|-1}\sum \limits _{{\mathbf {j}}\in I(\delta ,\nu )}f[{\mathbf {i}},{\mathbf {j}}]\sum \limits _{\varepsilon =1}^{|{\mathbf {n}}|-|{\mathbf {m}}|}\sum \limits _{{\mathbf {p}}\in I(\varepsilon ,\nu )}g^*[{\mathbf {e}}_{\alpha },{\mathbf {p}}]\,g[{\mathbf {p}}+{\mathbf {j}},{\mathbf {n}}]\\&= \sum \limits _{\gamma =|{\mathbf {m}}|}^{|{\mathbf {n}}|-1}\sum \limits _{{\mathbf {i}}\in I(\gamma ,\nu )}g^*[{\mathbf {m}},{\mathbf {i}}]\sum \limits _{\delta =|{\mathbf {m}}|}^{|{\mathbf {n}}|-1}\sum \limits _{{\mathbf {j}}\in I(\delta ,\nu )}f[{\mathbf {i}},{\mathbf {j}}]\sum \limits _{\varepsilon =1}^{|{\mathbf {n}}|-|{\mathbf {m}}|}\sum \limits _{{\mathbf {p}}\in I(\varepsilon ,\nu )}g^*[{\mathbf {e}}_{\alpha },{\mathbf {p}}]\\&\quad \times \sum \limits _{\sigma =\delta }^{|{\mathbf {n}}|-\varepsilon }\sum \limits _{{\mathbf {s}}\in I(\sigma ,\nu )}g[{\mathbf {j}},{\mathbf {s}}]\,g[{\mathbf {p}},{\mathbf {n}}-{\mathbf {s}}]=\sum \limits _{\gamma =|{\mathbf {m}}|}^{|{\mathbf {n}}|-1}\sum \limits _{{\mathbf {i}}\in I(\gamma ,\nu )}g^*[{\mathbf {m}},{\mathbf {i}}]\sum \limits _{\delta =|{\mathbf {m}}|}^{|{\mathbf {n}}|-1}\sum \limits _{{\mathbf {j}}\in I(\delta ,\nu )}f[{\mathbf {i}},{\mathbf {j}}]\\&\quad \times \sum \limits _{\sigma =\delta }^{|{\mathbf {n}}|-1}\sum \limits _{{\mathbf {s}}\in I(\sigma ,\nu )}g[{\mathbf {j}},{\mathbf {s}}]\sum \limits _{\varepsilon =1}^{|{\mathbf {n}}|-|{\mathbf {m}}|}\sum \limits _{{\mathbf {p}}\in I(\varepsilon ,\nu )}g^*[{\mathbf {e}}_{\alpha },{\mathbf {p}}]\,g[{\mathbf {p}},{\mathbf {n}}-{\mathbf {s}}]\\&= \sum \limits _{\gamma =|{\mathbf {m}}|}^{|{\mathbf {n}}|-1}\sum \limits _{{\mathbf {i}}\in I(\gamma ,\nu )}g^*[{\mathbf {m}},{\mathbf {i}}]\sum \limits _{\delta =|{\mathbf {m}}|}^{|{\mathbf {n}}|-1}\sum \limits _{{\mathbf {j}}\in I(\delta ,\nu )}f[{\mathbf {i}},{\mathbf {j}}]\,g[{\mathbf {j}},{\mathbf {n}}-{\mathbf {e}}_{\alpha }]=h[{\mathbf {m}},{\mathbf {n}}-{\mathbf {e}}_{\alpha }]. \end{aligned}$$

Consequently, the elements of ${\mathbf {H}}$ satisfy definition (12), i.e. ${\mathbf {H}}\in C^{\nu }(A)$.

Step 2. Let ${\mathbf {H}}={\mathbf {G}}^{-1}\,\dfrac{d'}{dt}\,{\mathbf {G}}$. We prove that ${\mathbf {H}}\in C^{\nu }(A)$. Let the superscript point denote differentiation $\dfrac{d}{dt}$. Here, we have:

$$\begin{aligned} h[{\mathbf {m}}+{\mathbf {e}}_{\alpha },{\mathbf {n}}]&= \sum \limits _{\gamma =|{\mathbf {m}}|+1}^{|{\mathbf {n}}|}\sum \limits _{{\mathbf {k}}\in I(\gamma ,\nu )}g^*[{\mathbf {m}}+{\mathbf {e}}_{\alpha },{\mathbf {k}}]\,\dot{g}[{\mathbf {k}},{\mathbf {n}}]\\&= \sum \limits _{\gamma =|{\mathbf {m}}|+1}^{|{\mathbf {n}}|}\sum \limits _{{\mathbf {k}}\in I(\gamma ,\nu )}\dot{g}[{\mathbf {k}},{\mathbf {n}}]\sum \limits _{\delta =1}^{\gamma -|{\mathbf {m}}|}\sum \limits _{{\mathbf {p}}\in I(\delta ,\nu )}g^*[{\mathbf {e}}_{\alpha },{\mathbf {p}}]\,g^*[{\mathbf {m}},{\mathbf {k}}-{\mathbf {p}}]\\&= \sum \limits _{\delta =1}^{|{\mathbf {n}}|-|{\mathbf {m}}|}\sum \limits _{{\mathbf {p}}\in I(\delta ,\nu )}g^*[{\mathbf {e}}_{\alpha },{\mathbf {p}}]\sum \limits _{\gamma =|{\mathbf {m}}|+\delta }^{|{\mathbf {n}}|}\sum \limits _{{\mathbf {k}}\in I(\gamma ,\nu )}\dot{g}[{\mathbf {k}},{\mathbf {n}}]\,g^*[{\mathbf {m}},{\mathbf {k}}-{\mathbf {p}}]\\&= \sum \limits _{\delta =1}^{|{\mathbf {n}}|-|{\mathbf {m}}|}\sum \limits _{{\mathbf {p}}\in I(\delta ,\nu )}g^*[{\mathbf {e}}_{\alpha },{\mathbf {p}}]\sum \limits _{\gamma =|{\mathbf {m}}|}^{|{\mathbf {n}}|-1}\sum \limits _{{\mathbf {l}}\in I(\gamma ,\nu )}\dot{g}[{\mathbf {l}}+{\mathbf {p}},{\mathbf {n}}]\,g^*[{\mathbf {m}},{\mathbf {l}}]\\&= \sum \limits _{\delta =1}^{|{\mathbf {n}}|-|{\mathbf {m}}|}\sum \limits _{{\mathbf {p}}\in I(\delta ,\nu )}g^*[{\mathbf {e}}_{\alpha },{\mathbf {p}}]\sum \limits _{\gamma =|{\mathbf {m}}|}^{|{\mathbf {n}}|-1}\sum \limits _{{\mathbf {l}}\in I(\gamma ,\nu )}g^*[{\mathbf {m}},{\mathbf {l}}]\\&\quad \times \sum \limits _{\sigma =\gamma }^{|{\mathbf {n}}|-\delta }\sum \limits _{{\mathbf {q}}\in I(\sigma ,\nu )}\left( \dot{g}[{\mathbf {l}},{\mathbf {q}}]\,g[{\mathbf {p}},{\mathbf {n}}-{\mathbf {q}}]+g[{\mathbf {l}},{\mathbf {q}}]\,\dot{g}[{\mathbf {p}},{\mathbf {n}}-{\mathbf {q}}]\right) \\&= \sum \limits _{\gamma =|{\mathbf {m}}|}^{|{\mathbf {n}}|-1}\sum \limits _{{\mathbf {l}}\in I(\gamma ,\nu )}g^*[{\mathbf {m}},{\mathbf {l}}]\sum \limits _{\sigma =\gamma }^{|{\mathbf {n}}|-1}\sum \limits _{{\mathbf {q}}\in I(\sigma ,\nu )}\dot{g}[{\mathbf {l}},{\mathbf {q}}]\sum \limits _{\delta =1}^{|{\mathbf {n}}|-|{\mathbf {m}}|}\sum \limits _{{\mathbf {p}}\in I(\delta ,\nu )}g^*[{\mathbf {e}}_{\alpha },{\mathbf {p}}]\,g[{\mathbf {p}},{\mathbf {n}}-{\mathbf {q}}]\\&\quad +\sum \limits _{\delta =1}^{|{\mathbf {n}}|-|{\mathbf {m}}|}\sum \limits _{{\mathbf {p}}\in I(\delta ,\nu )}g^*[{\mathbf {e}}_{\alpha },{\mathbf {p}}]\sum \limits _{\sigma =|{\mathbf {m}}|}^{|{\mathbf {n}}|-\delta }\sum \limits _{{\mathbf {q}}\in I(\sigma ,\nu )}\dot{g}[{\mathbf {p}},{\mathbf {n}}-{\mathbf {q}}]\sum \limits _{\gamma =|{\mathbf {m}}|}^{|{\mathbf {n}}|-1}\sum \limits _{{\mathbf {l}}\in I(\gamma ,\nu )}g^*[{\mathbf {m}},{\mathbf {l}}]\,g[{\mathbf {l}},{\mathbf {q}}]\\&= \sum \limits _{\gamma =|{\mathbf {m}}|}^{|{\mathbf {n}}|-1}\sum \limits _{{\mathbf {l}}\in I(\gamma ,\nu )}g^*[{\mathbf {m}},{\mathbf {l}}]\,\dot{g}[{\mathbf {l}},{\mathbf {n}}-{\mathbf {e}}_{\alpha }]+\sum \limits _{\delta =1}^{|{\mathbf {n}}|-|{\mathbf {m}}|}\sum \limits _{{\mathbf {p}}\in I(\delta ,\nu )}g^*[{\mathbf {e}}_{\alpha },{\mathbf {p}}]\,\dot{g}[{\mathbf {p}},{\mathbf {n}}-{\mathbf {m}}]\\&= h[{\mathbf {m}},{\mathbf {n}}-{\mathbf {e}}_{\alpha }]+h[{\mathbf {e}}_{\alpha },{\mathbf {n}}-{\mathbf {m}}]. \end{aligned}$$

The elements of **H** satisfy definition (12), i.e., ${\mathbf {H}}\in C^{\nu }(A)$.

Step 3. It is easy to see that if ${\mathbf {H}}_1,\,{\mathbf {H}}_2\in C^{\nu }(A)$, then ${\mathbf {H}}_1+{\mathbf {H}}_2\in C^{\nu }(A)$. Consequently, $A_{\mathbf {G}}:C^{\nu }(A)\rightarrow C^{\nu }(A)$.  $\square$

### Proof of Proposition 7

Step 1. Let ${\mathbf {G}}\in W^{\nu }(A)$. We prove that:

13 $$\dot{g}[{\mathbf {m}},{\mathbf {n}}] =\sum \limits _{\alpha =1}^{\nu }m_{\alpha }\sum \limits _{\varepsilon =1}^{|{\mathbf {n}}|-|{\mathbf {m}}|+1}\sum \limits _{{\mathbf {l}}\in I(\varepsilon ,\nu )}\dot{g}[{\mathbf {e}}_{\alpha },{\mathbf {l}}]\,g[{\mathbf {m}}-{\mathbf {e}}_{\alpha },{\mathbf {n}}-{\mathbf {l}}].$$

Let ${\mathbf {m}}\in I(1,\nu ),\,{\mathbf {m}}={\mathbf {e}}_{\gamma },\,\gamma =1,\ldots ,\nu$. Setting $g[{\mathbf {0}},{\mathbf {0}}]=1$, one obtains that:

$$\sum \limits _{\varepsilon =1}^{|{\mathbf {n}}|}\sum \limits _{{\mathbf {l}}\in I(\varepsilon ,\nu )}\dot{g}[{\mathbf {e}}_{\gamma },{\mathbf {l}}] \,g[{\mathbf {0}},{\mathbf {n}}-{\mathbf {l}}]=\dot{g}[{\mathbf {e}}_{\gamma },{\mathbf {n}}].$$

These calculations prove (13) for $|{\mathbf {m}}|=1$. Let $|{\mathbf {m}}|=2,\,{\mathbf {m}}={\mathbf {e}}_{\gamma }+{\mathbf {e}}_{\delta },$ where $\gamma ,\,\delta =1,\ldots ,\nu$. Then:

$$\begin{aligned}&\sum \limits _{\alpha =1}^{\nu }m_{\alpha }\sum \limits _{\varepsilon =1}^{|{\mathbf {n}}|-|{\mathbf {m}}|+1}\sum \limits _{{\mathbf {l}}\in I(\varepsilon ,\nu )}\dot{g}[{\mathbf {e}}_{\alpha },{\mathbf {l}}]\,g[{\mathbf {m}}-{\mathbf {e}}_{\alpha },{\mathbf {n}}-{\mathbf {l}}]\\&\quad =\sum \limits _{\sigma =1}^{|{\mathbf {n}}|-1}\sum \limits _{{\mathbf {l}}\in I(\sigma ,\nu )}\dot{g}[{\mathbf {e}}_{\gamma },{\mathbf {l}}]\,g[{\mathbf {e}}_{\delta },{\mathbf {n}}-{\mathbf {l}}]+\sum \limits _{\zeta =1}^{|{\mathbf {n}}|-1}\sum \limits _{{{\mathbf {j}}}\in I(\zeta ,\nu )}\dot{g}[{\mathbf {e}}_{\delta },{\mathbf {j}}]\,g[{\mathbf {e}}_{\gamma },{\mathbf {n}}-{\mathbf {j}}]\\&\quad =\sum \limits _{\sigma =1}^{|{\mathbf {n}}|-1}\sum \limits _{{\mathbf {l}}\in I(\sigma ,\nu )}\dot{g}[{\mathbf {e}}_{\gamma },{\mathbf {l}}]\,g[{\mathbf {e}}_{\delta },{\mathbf {n}}-{\mathbf {l}}]+\sum \limits _{\zeta =1}^{|{\mathbf {n}}|-1}\sum \limits _{{\mathbf {k}}\in I(\zeta ,\nu )}\dot{g}[{\mathbf {e}}_{\delta },{\mathbf {n}}-{\mathbf {k}}]\,g[{\mathbf {e}}_{\gamma },{\mathbf {k}}]\\&\quad =\sum \limits _{\sigma =1}^{|{\mathbf {n}}|-1}\sum \limits _{{\mathbf {l}}\in I(\sigma ,\nu )}\left( \dot{g}[{\mathbf {e}}_{\gamma },{\mathbf {l}}]\,g[{\mathbf {e}}_\delta ,{\mathbf {n}}-{\mathbf {l}}]+\dot{g}[{\mathbf {e}}_{\delta },{\mathbf {n}}-{\mathbf {l}}]\,g[{\mathbf {e}}_{\gamma },{\mathbf {l}}]\right) =\dot{g}[{\mathbf {e}}_{\gamma }+{\mathbf {e}}_{\delta },{\mathbf {n}}]. \end{aligned}$$

These expressions prove (13) for $|{\mathbf {m}}|=2$. Suppose by induction that (13) holds for $|{\mathbf {m}}|\ge 2$. Then, for $\beta =1,\ldots ,\nu$, one obtains:

$$\begin{aligned} \dot{g}[{\mathbf {m}}+{\mathbf {e}}_{\beta },{\mathbf {n}}]&= \sum \limits _{\gamma =1}^{|{\mathbf {n}}|-|{\mathbf {m}}|}\sum \limits _{{\mathbf {k}}\in I(\gamma ,\nu )}\left( \dot{g}[{\mathbf {e}}_{\beta },{\mathbf {k}}]\,g[{\mathbf {m}},{\mathbf {n}}-{\mathbf {k}}]+g[{\mathbf {e}}_{\beta },{\mathbf {k}}]\,\dot{g}[{\mathbf {m}},{\mathbf {n}}-{\mathbf {k}}]\right) \\&= \sum \limits _{\gamma =1}^{|{\mathbf {n}}|-|{\mathbf {m}}|} \sum \limits _{{\mathbf {k}}\in I(\gamma ,\nu )} g[{\mathbf {e}}_{\beta },{\mathbf {k}}] \sum \limits _{\alpha =1}^{\nu }m_{\alpha }\sum \limits _{\varepsilon =1}^{|{\mathbf {n}}|-\gamma +|{\mathbf {m}}|+1}\sum \limits _{{\mathbf {l}}\in I(\varepsilon ,\nu )} \dot{g}[{\mathbf {e}}_{\alpha },{\mathbf {l}}] \,g[{\mathbf {m}}-{\mathbf {e}}_{\alpha },{\mathbf {n}}-{\mathbf {k}} -{\mathbf {l}}]\\&\quad +\sum \limits _{\gamma =1}^{|{\mathbf {n}}|-|{\mathbf {m}}|}\sum \limits _{{\mathbf {k}}\in I(\gamma ,\nu )}\dot{g}[{\mathbf {e}}_{\beta },{\mathbf {k}}]\,g[{\mathbf {m}}, {\mathbf {n}}-{\mathbf {k}}]=\sum \limits _{\alpha =1}^{\nu }m_{\alpha }\sum \limits _{\varepsilon =1}^{|{\mathbf {n}}|-|{\mathbf {m}}|}\sum \limits _{{\mathbf {l}}\in I(\varepsilon ,\nu )}\dot{g}[{\mathbf {e}}_{\alpha },{\mathbf {l}}]\\&\quad \times \sum \limits _{\gamma =1}^{|{\mathbf {n}}|-\varepsilon -|{\mathbf {m}}|+1}\sum \limits _{{\mathbf {k}}\in I(\gamma ,\nu )}g[{\mathbf {e}}_{\beta },{\mathbf {k}}]\,g[{\mathbf {m}} -{\mathbf {e}}_{\alpha },{\mathbf {n}}-{\mathbf {l}}-{\mathbf {k}}]\\&\quad +\sum \limits _{\gamma =1}^{|{\mathbf {n}}|-|{\mathbf {m}}|}\sum \limits _{{\mathbf {k}} \in I(\gamma ,\nu )}\dot{g}[{\mathbf {e}}_{\beta },{\mathbf {k}}]\,g[{\mathbf {m}},{\mathbf {n}}-{\mathbf {k}}]\\&= \sum \limits _{\alpha =1}^{\nu }m_{\alpha }\sum \limits _{\varepsilon =1}^{|{\mathbf {n}}|-|{\mathbf {m}}|}\sum \limits _{{\mathbf {l}}\in I(\varepsilon ,\nu )}\dot{g}[{\mathbf {e}}_{\alpha },{\mathbf {l}}]\,g[{\mathbf {m}}-{\mathbf {e}}_{\alpha }+{\mathbf {e}}_{\beta },{\mathbf {n}}-{\mathbf {l}}]\\&\quad +\sum \limits _{\gamma =1}^{|{\mathbf {n}}|-|{\mathbf {m}}|}\sum \limits _{{\mathbf {l}}\in I(\gamma ,\nu )}\dot{g}[{\mathbf {e}}_{\beta },{\mathbf {l}}]\,g[{\mathbf {m}},{\mathbf {n}}-{\mathbf {l}}]\\&= \sum \limits _{\alpha =1}^{\nu }(m_{\alpha }+\delta _{\alpha }^{\beta })\sum \limits _{\varepsilon =1}^{|{\mathbf {n}}|-|{\mathbf {m}}|}\sum \limits _{{\mathbf {l}}\in I(\varepsilon ,\nu )}\dot{g}[{\mathbf {e}}_{\alpha },{\mathbf {l}}]\,g[{\mathbf {m}}-{\mathbf {e}}_{\alpha }+{\mathbf {e}}_{\beta },{\mathbf {n}}-{\mathbf {l}}] \end{aligned}$$

where $\delta _{\alpha }^{\beta }$ is the Kronecker symbol. These calculations prove (13).

Step 2. We prove the equality of Carleman matrices:

14 $$\varphi ({\mathbf {J}}_{\mathbf {g}}^{-1}\cdot ({\mathbf {f}}\circ {\mathbf {g}}))=(\psi ({\mathbf {g}}))^{-1}\varphi ({\mathbf {f}})\psi ({\mathbf {g}}).$$

To prove this equality, it is sufficient to show that the leading $\nu$ rows of matrices are equal. Let ${\mathbf {X}}={\mathbf {g}}(t, {{\mathbf {Y}}}),\,{\mathbf {w}}(t,{{\mathbf {Y}}}) ={\mathbf {J}}_{\mathbf {g}}^{-1}(t,{{\mathbf {Y}}})\cdot ({\mathbf {f}}\circ {\mathbf {g}})(t,{\mathbf {Y}})$. Then:

$${\mathbf {J}}_{\mathbf {g}}^{-1}(t,{\mathbf {Y}}) =\bigl .{\mathbf {J}}_{{\mathbf {g}}^{-1}}(t,{\mathbf {X}})\bigr |_{{\mathbf {X}}={\mathbf {g}}(t,{\mathbf {Y}})}=({\mathbf {J}}_{{\mathbf {g}}^{-1}}\circ {\mathbf {g}})(t,{\mathbf {Y}}).$$

It follows that:

$$\begin{aligned} w_{\beta }&= \left( (grad(g_{\beta }^{-1})\right) \circ {\mathbf {g}})(t,{\mathbf {Y}})\cdot ({\mathbf {f}}\circ {\mathbf {g}})(t,{\mathbf {Y}})\\&= \sum \limits _{\alpha =1}^{\nu }\sum \limits _{\gamma =1}^{\infty }\sum \limits _{{\mathbf {n}}\in I(\gamma ,\nu )}n_{\alpha }g^*[{\mathbf {e}}_{\beta },{\mathbf {n}}]\sum \limits _{\delta =\gamma -1}^{\infty }\sum \limits _{{\mathbf {m}}\in I(\delta ,\nu )}g[{\mathbf {n}}-{\mathbf {e}}_{\alpha },{\mathbf {m}}]\,y[{\mathbf {m}}]\\&\quad \times \sum \limits _{\varepsilon =1}^{\infty }\sum \limits _{{\mathbf {k}}\in I(\varepsilon ,\nu )}f[{\mathbf {e}}_{\alpha },{\mathbf {k}}]\sum \limits _{\sigma =\varepsilon }^{\infty }\sum \limits _{{\mathbf {i}}\in I(\sigma ,\nu )}g[{\mathbf {k}},{\mathbf {i}}]\,y[{\mathbf {i}}]\\&= \sum \limits _{\delta =1}^{\infty }\sum \limits _{{\mathbf {l}}\in I(\delta ,\nu )}\sum \limits _{\alpha =1}^{\nu }\sum \limits _{\gamma =1}^{\infty }\sum \limits _{{\mathbf {n}}\in I(\gamma ,\nu )}\sum \limits _{\varepsilon =1}^{\infty }\sum \limits _{{\mathbf {k}}\in I(\varepsilon ,\nu )}n_{\alpha }g^*[{\mathbf {e}}_{\beta },{\mathbf {n}}]\,f[{\mathbf {e}}_{\alpha },{\mathbf {k}}]\\&\quad \times \left( \sum \limits _{\sigma =\varepsilon }^{\delta }\sum \limits _{{\mathbf {i}}\in I(\sigma ,\nu )}g[{\mathbf {n}}-{\mathbf {e}}_{\alpha },{\mathbf {l}}-{\mathbf {i}}]\,g[{\mathbf {k}},{\mathbf {i}}]\right) y[{\mathbf {l}}]=\sum \limits _{\delta =1}^{\infty }\sum \limits _{{\mathbf {l}}\in I(\delta ,\nu )}\sum \limits _{\alpha =1}^{\nu }\sum \limits _{\gamma =1}^{\infty }\sum \limits _{{\mathbf {n}}\in I(\gamma ,\nu )}\\&\quad \times \sum \limits _{\varepsilon =1}^{\infty }\sum \limits _{{\mathbf {k}}\in I(\varepsilon ,\nu )}n_{\alpha }g^*[{\mathbf {e}}_{\beta },{\mathbf {n}}]\,f[{\mathbf {e}}_{\alpha },{\mathbf {k}}]\,g[{\mathbf {k}}+{\mathbf {n}}-{\mathbf {e}}_{\alpha },{\mathbf {l}}]\,y[{\mathbf {l}}]\\&= \sum \limits _{\delta =1}^{\infty }\sum \limits _{{\mathbf {l}}\in I(\delta ,\nu )}\sum \limits _{\gamma =1}^{\infty }\sum \limits _{{\mathbf {n}}\in I(\gamma ,\nu )}\sum \limits _{\varepsilon =1}^{\delta }\sum \limits _{{\mathbf {j}}\in I(\varepsilon ,\nu )}g^*[{\mathbf {e}}_{\beta },{\mathbf {n}}]\\&\quad \times \left( \sum \limits _{\alpha =1}^{\nu }n_{\alpha }f[{\mathbf {e}}_{\alpha },{\mathbf {j}}-{\mathbf {n}}+{\mathbf {e}}_{\alpha }]\right) g[{\mathbf {j}},{\mathbf {l}}]\,y[{\mathbf {l}}]\\&= \sum \limits _{\delta =1}^{\infty }\sum \limits _{{\mathbf {l}}\in I(\delta ,\nu )}\left( \sum \limits _{\gamma =1}^{\delta }\sum \limits _{{\mathbf {n}}\in I(\gamma ,\nu )}g^*[{\mathbf {e}}_{\beta },{\mathbf {n}}]\sum \limits _{\varepsilon =\gamma }^{\delta }\sum \limits _{{\mathbf {j}}\in I(\varepsilon ,\nu )}f[{\mathbf {n}},{\mathbf {j}}]\,g[{\mathbf {j}},{\mathbf {l}}]\right) y[{\mathbf {l}}]. \end{aligned}$$

This mathematics proves (14).

Step 3. Now, we prove the equality of Carleman matrices:

15 $$\varphi \left( {\mathbf {J}}_{\mathbf {g}}^{-1}\dfrac{\partial {\mathbf {g}}}{\partial t}\right) =(\psi ({\mathbf {g}}))^{-1}\dfrac{d'}{dt}\,\psi ({\mathbf {g}}).$$

Let: ${\mathbf {X}}={\mathbf {g}}(t,{\mathbf {Y}}),\,{\mathbf {u}}={\mathbf {J}}_{\mathbf {g}}^{-1}\dfrac{\partial {\mathbf {g}}}{\partial t}$. Then, we have:

$$\begin{aligned} u_{\beta }&= (grad(g_{\beta }^{-1})\circ {\mathbf {g}})(t,{\mathbf {Y}})\times \dfrac{\partial {\mathbf {g}}(t,{\mathbf {Y}})}{\partial t}\\&= \sum \limits _{\alpha =1}^{\nu }\sum \limits _{\gamma =1}^{\infty }\sum \limits _{{\mathbf {m}}\in I(\gamma ,\nu )}m_{\alpha }g^*[{\mathbf {e}}_{\beta },{\mathbf {m}}]\sum \limits _{\delta =\gamma -1}^{\infty }\sum \limits _{{\mathbf {k}}\in I(\delta ,\nu )}g[{\mathbf {m}}-{\mathbf {e}}_{\alpha },{\mathbf {k}}]\,y[{\mathbf {k}}]\\&\quad \times \sum \limits _{\varepsilon =1}^{\infty }\sum \limits _{{\mathbf {n}}\in I(\varepsilon ,\nu )}\dot{g}[{\mathbf {e}}_{\alpha },{\mathbf {n}}]\,y[{\mathbf {n}}]=\sum \limits _{\delta =1}^{\infty }\sum \limits _{{\mathbf {l}}\in I(\delta ,\nu )}\sum \limits _{\gamma =1}^{\infty }\sum \limits _{{\mathbf {m}}\in I(\gamma ,\nu )}g^*[{\mathbf {e}}_{\beta },{\mathbf {m}}]\\&\quad \times \sum \limits _{\alpha =1}^{\nu }m_{\alpha }\sum \limits _{\varepsilon =1}^{\delta -\gamma +1}\sum \limits _{{\mathbf {n}}\in I(\varepsilon ,\nu )}\dot{g}[{\mathbf {e}}_{\alpha },{\mathbf {n}}]\,g[{\mathbf {m}}-{\mathbf {e}}_{\alpha },{\mathbf {l}}-{n}]\,y[{\mathbf {l}}]. \end{aligned}$$

Hence, by (13):

$$u_{\beta }=\sum \limits _{\delta =1}^{\infty }\sum \limits _{{\mathbf {l}}\in I(\delta ,\nu )}\left( \sum \limits _{\gamma =1}^{\delta }\sum \limits _{{\mathbf {m}}\in I(\gamma ,\nu )}g^*[{\mathbf {e}}_{\beta },{\mathbf {m}}]\,\dot{g}[{\mathbf {m}},{\mathbf {l}}]\right) y[{\mathbf {l}}]$$

which proves (15).

Step 4. Since $\varphi$ is a linear mapping, by (14) and (15):

$$\begin{aligned} (\varphi \circ a_{\mathbf {g}})({\mathbf {f}})&= \varphi ({\mathbf {J}}_{\mathbf {g}}^{-1}({\mathbf {f}}\circ {\mathbf {g}}))-\varphi ({\mathbf {J}}_{\mathbf {g}}^{-1}\,\dfrac{\partial {\mathbf {g}}}{\partial t})\\&= (\psi ({\mathbf {g}}))^{-1}\varphi ({\mathbf {f}})\psi ({\mathbf {g}})-(\psi ({\mathbf {g}}))^{-1}\,\dfrac{d'}{dt}\,\psi ({\mathbf {g}})\\&= (A_{\psi ({\mathbf {g}})}\circ \varphi )({\mathbf {f}}). \end{aligned}$$

These calculations prove the proposition.  $\square$

## Example

In this section, we give an example of the algorithm introduced above. Consider the following system of ordinary differential equations with quasi-periodic coefficients with a frequency set ${\varvec{\omega }}=(1,\pi )$:

$$\left\{ \begin{array}{l} \dfrac{dz_{1}}{dt}=i z_1 + a z_{1}{z}_{2}+2{z}_{1}^{2}{z}_{2},\\ \dfrac{dz_{2}}{dt}=-i z_2 + \overline{a} z_{1}{z}_{2}+2{z}_{1}{z}_{2}^{2}, \end{array} \right.$$

where ${\mathbf {Z}}=(z_1,z_2) \in {\mathbb {C}}^2, z_1=\overline{z_2}$, $a=1+e^{it}+e^{i{\pi }t}$. We shall compute a third order normal form. For calculating the Carleman matrix ${\mathbf {F}}$ we can use either the formula (5) or the direct differentiation of monomials:

$$\begin{aligned} \dfrac{d}{dt}\,\left( z_1^2\right)&= 2i z_1^2 + 2 a z_{1}^2 z_{2}+ O\left( \Vert {\mathbf {Z}}\Vert ^4\right) ,\\ \dfrac{d}{dt}\,\left( z_1 z_2\right)&= \overline{a} z_1^{2}z_2 + a z_{1} z_{2}^{2}+O\left( \Vert {\mathbf {Z}}\Vert ^4\right) ,\\ \dfrac{d}{dt}\,\left( z_2^2\right)&= -2i z_2^2 + 2 \overline{a} z_{1} z_{2}^2+ O\left( \Vert {\mathbf {Z}}\Vert ^4\right) ,\\ \dfrac{d}{dt}\,\left( z_1^3\right)&= 3i z_1^3 + O\left( \Vert {\mathbf {Z}}\Vert ^4\right) ,\\ \dfrac{d}{dt}\,\left( z_1^2 z_2\right)&= i z_1^2 z_2 + O\left( \Vert {\mathbf {Z}}\Vert ^4\right) ,\\ \dfrac{d}{dt}\,\left( z_1 z_2^2\right)&= -i z_1 z_2^2 + O\left( \Vert {\mathbf {Z}}\Vert ^4\right) ,\\ \dfrac{d}{dt}\,\left( z_2^3\right)&= -3i z_2^3 + O\left( \Vert {\mathbf {Z}}\Vert ^4\right) . \end{aligned}$$

Then we have the following Carleman matrix **F**:

$$\begin{aligned} \begin{array}{ccccccccccc} {\mathbf {F}} &\quad &\quad 1 &\quad 2 &\quad 3 &\quad 4 &\quad 5 &\quad 6 &\quad 7 &\quad 8 &\quad 9 \\ &\quad &\quad (1,0)&\quad (0,1)&\quad (2,0)&\quad (1,1) &\quad (0,2)&\quad (3,0)&\quad (2,1) &\quad (1,2) &\quad (0,3) \\ 1&\quad (1,0) &\quad i &\quad &\quad &\quad a &\quad &\quad &\quad 2 &\quad &\quad \\ 2&\quad (0,1) &\quad &\quad -i &\quad &\quad \overline{a}&\quad &\quad &\quad &\quad 2 &\quad \\ 3&\quad (2,0) &\quad &\quad &\quad 2i &\quad &\quad &\quad &\quad 2a &\quad &\quad \\ 4&\quad (1,1) &\quad &\quad &\quad &\quad 0 &\quad &\quad &\quad \overline{a}&\quad a &\quad \\ 5&\quad (0,2) &\quad &\quad &\quad &\quad &\quad -2i &\quad &\quad &\quad 2\overline{a}&\quad \\ 6&\quad (3,0) &\quad &\quad &\quad &\quad &\quad &\quad 3i &\quad &\quad &\quad \\ 7&\quad (2,1) &\quad &\quad &\quad &\quad &\quad &\quad &\quad i &\quad &\quad \\ 8&\quad (1,2) &\quad &\quad &\quad &\quad &\quad &\quad &\quad &\quad -i &\quad \\ 9&\quad (0,3) &\quad &\quad &\quad &\quad &\quad &\quad &\quad &\quad &\quad -3i. \\ \end{array} \end{aligned}$$

The elements of the Weierstrass matrix ${\mathbf {G}}$ of normalizing transformation ${\mathbf {Z}}=g({\mathbf {Y}})$, ${\mathbf {Y}}=(y_1,y_2)$ and the normal form ${\mathbf {H}}$ are calculated in the order (8). The elements of the leading two rows of ${\mathbf {G}}$ and ${\mathbf {H}}$ are calculated by (9) and (10), respectively. One obtains the elements of ${\mathbf {G}}$ and ${\mathbf {H}}$ below the second row by (3) and (5), respectively.

The Weierstrass matrix ${\mathbf {G}}$ of normalizing transformation takes the form:

$$\begin{aligned}{}\begin{array}[t]{ccccccccccc} {\mathbf {G}} &\quad &\quad1 &\quad 2 &\quad 3 &\quad 4 &\quad 5 &\quad 6 &\quad 7 &\quad 8 &\quad 9 \\ &\quad &\quad (1,0)&\quad (0,1)&\quad (2,0)&\quad (1,1) &\quad (0,2)&\quad (3,0)&\quad (2,1) &\quad (1,2) &\quad (0,3) \\ 1&\quad (1,0) &\quad 1 &\quad &\quad &\quad b &\quad &\quad &\quad c &\quad d &\quad \\ 2&\quad (0,1) &\quad &\quad 1 &\quad &\quad \overline{b}&\quad &\quad &\quad \overline{d}&\quad \overline{c} &\quad \\ 3&\quad (2,0) &\quad &\quad &\quad 1 &\quad &\quad &\quad &\quad 2b &\quad &\quad \\ 4&\quad (1,1) &\quad &\quad &\quad &\quad 1 &\quad &\quad &\quad \overline{b}&\quad b &\quad \\ 5&\quad (0,2) &\quad &\quad &\quad &\quad &\quad 1 &\quad &\quad &\quad 2\overline{b}&\quad \\ 6&\quad (3,0) &\quad &\quad &\quad &\quad &\quad &\quad 1 &\quad &\quad &\quad \\ 7&\quad (2,1) &\quad &\quad &\quad &\quad &\quad &\quad &\quad 1 &\quad &\quad \\ 8&\quad (1,2) &\quad &\quad &\quad &\quad &\quad &\quad &\quad &\quad 1 &\quad \\ 9&\quad (0,3) &\quad &\quad &\quad &\quad &\quad &\quad &\quad &\quad &\quad 1, \end{array} \end{aligned}$$

where *b*, *c*, *d* are as follows:

$$\begin{aligned} b&=i - \frac{i}{\pi - 1}e^{i\pi t}, \quad d = - \frac{1}{2} + \frac{1}{\pi - 1}e^{i\pi t} - \frac{1}{2 (\pi - 1)^2}e^{2i\pi t},\\ c& = e^{-it} - e^{it} - \frac{1}{\pi (\pi - 1)}e^{-i\pi t} - \frac{1}{\pi }e^{i\pi t} - \frac{1}{(\pi - 1)^2}e^{it - i\pi t} + \frac{1}{(\pi - 1)^2}e^{i\pi t - it}. \end{aligned}$$

The Weierstrass matrix ${\mathbf {G^{-1}}}$ of the inverse of the normalizing transformation ${\mathbf {Y}}=g^{-1}({\mathbf {Z}})$ takes the form:

$$\begin{aligned} \begin{array}{ccccccccccc} {\mathbf {G^{-1}}} &\quad &\quad 1 &\quad 2 &\quad 3 &\quad 4 &\quad 5 &\quad 6 &\quad 7 &\quad 8 &\quad 9\\ &\quad &\quad (1,0)&\quad (0,1)&\quad (2,0)&\quad (1,1) &\quad (0,2)&\quad (3,0)&\quad (2,1) &\quad (1,2) &\quad (0,3)\\ 1&\quad (1,0) &\quad 1 &\quad &\quad &\quad -b &\quad &\quad &\quad b\overline{b}-c &\quad b^2-d &\quad \\ 2&\quad (0,1) &\quad &\quad 1 &\quad &\quad -\overline{b}&\quad &\quad &\quad \overline{b}^2-\overline{d} &\quad b\overline{b}-\overline{c}&\quad \\ 3&\quad (2,0) &\quad &\quad &\quad 1 &\quad &\quad &\quad &\quad -2b &\quad &\quad \\ 4&\quad (1,1) &\quad &\quad &\quad &\quad 1 &\quad &\quad &\quad -\overline{b} &\quad -b &\quad \\ 5&\quad (0,2) &\quad &\quad &\quad &\quad &\quad 1 &\quad &\quad &\quad -2\overline{b} &\quad \\ 6&\quad (3,0) &\quad &\quad &\quad &\quad &\quad &\quad 1 &\quad &\quad &\quad \\ 7&\quad (2,1) &\quad &\quad &\quad &\quad &\quad &\quad &\quad 1 &\quad &\quad \\ 8&\quad (1,2) &\quad &\quad &\quad &\quad &\quad &\quad &\quad &\quad 1 &\quad \\ 9&\quad (0,3) &\quad &\quad &\quad &\quad &\quad &\quad &\quad &\quad &\quad 1. \end{array} \end{aligned}$$

The normal form ${\mathbf {H}}$ of the Carleman matrix ${\mathbf {F}}$ takes the form:

$$\begin{aligned} \begin{array}{ccccccccccc} {\mathbf {H}} &\quad &\quad 1 &\quad 2 &\quad 3 &\quad 4 &\quad 5 &\quad 6 &\quad 7 &\quad 8 &\quad 9 \\ &\quad &\quad (1,0)&\quad (0,1)&\quad (2,0)&\quad (1,1) &\quad (0,2)&\quad (3,0)&\quad (2,1) &\quad (1,2) &\quad (0,3) \\ 1&\quad (1,0) &\quad i &\quad &\quad &\quad p &\quad &\quad &\quad q &\quad &\quad \\ 2&\quad (0,1) &\quad &\quad -i &\quad &\quad \overline{p}&\quad &\quad &\quad &\quad \overline{q} &\quad \\ 3&\quad (2,0) &\quad &\quad &\quad 2i &\quad &\quad &\quad &\quad 2p &\quad &\quad \\ 4&\quad (1,1) &\quad &\quad &\quad &\quad 0 &\quad &\quad &\quad \overline{p}&\quad p &\quad \\ 5&\quad (0,2) &\quad &\quad &\quad &\quad &\quad -2i &\quad &\quad &\quad 2\overline{p}&\quad \\ 6&\quad (3,0) &\quad &\quad &\quad &\quad &\quad &\quad 3i &\quad &\quad &\quad \\ 7&\quad (2,1) &\quad &\quad &\quad &\quad &\quad &\quad &\quad i &\quad &\quad \\ 8&\quad (1,2) &\quad &\quad &\quad &\quad &\quad &\quad &\quad &\quad -i &\quad \\ 9&\quad (0,3) &\quad &\quad &\quad &\quad &\quad &\quad &\quad &\quad &\quad -3i, \end{array} \end{aligned}$$

where *p*, *q* are as follows: $p=e^{i t}, \quad q = 2 - \frac{\pi -2}{\pi - 1}\,i$.

For example, consider the calculation of the elements $g_{1,7}=g[(1,0),(2,1)], h_{1,7}=h[(1,0),(2,1)]$. From the proof of the theorem we have:

$$\begin{aligned} \dfrac{d}{dt}g_{1,7}&= \left( f_{1,1} - f_{7,7}\right) g_{1,7} + \left( \sum \limits _{\gamma =2}^{7}f_{1,\gamma }g_{\gamma ,7}-\sum \limits _{\gamma =1}^{6}g_{1,\gamma }h_{\gamma ,7}\right) ,\\&\quad \left( f_{1,1} - f_{7,7}\right) g_{1,7} + \left( \sum \limits _{\gamma =2}^{7}f_{1,\gamma }g_{\gamma ,7}-\sum \limits _{\gamma =1}^{6}g_{1,\gamma }h_{\gamma ,7}\right) \\&= f_{1,4}g_{4,7} + f_{1,7}g_{7,7} - g_{1,1}h_{1,7} - g_{1,4}h_{4,7} = a\overline{b} + 2 - h_{1,7} - b\overline{p} \\&= 2 - \frac{\pi -2}{\pi - 1}\,i - i e^{-i t} - i e^{i t} + \frac{i}{\pi - 1}e^{-i\pi t} - i e^{i \pi t} + \frac{i}{\pi -1}e^{it-i\pi t}\\&\quad +\frac{i}{\pi - 1}e^{i\pi t - it} - h_{1,7}. \end{aligned}$$

One can see, that the harmonic $2 - \frac{\pi -2}{\pi - 1}\,i$ is resonant. In this case, *g*[(1, 0), (2, 1), (0, 0)] may be assigned an arbitrary value. Let $g[(1,0),(2,1),(0,0)]=0$. Then we have $h_{1,7} = 2 - \frac{\pi -2}{\pi - 1}\,i$. The coefficients of non-resonant harmonics in $g_{1,7}$ are calculated by (9):

$$g_{1,7}=e^{-it}-e^{it}-\frac{1}{\pi (\pi -1)}e^{-i\pi t}-\frac{1}{\pi }e^{i\pi t}-\frac{1}{(\pi -1)^2}e^{it-i\pi t}+\frac{1}{(\pi -1)^2}e^{i\pi t-it}.$$

Consider the calculation of the element $g_{5,8}=g[(0,2),(1,2)]$. From (3) or (11) we have:

$$\begin{aligned} g_{5,8}&= g[(0,2),(1,2)]=g[(0,1)+(0,1),(1,2)]\\&= \sum \limits _{\mu =1}^{2}\sum \limits _{{\mathbf {k}}\in I(\mu ,2)}g[(0,1),{\mathbf {k}}]\,g[(0,1),(1,2)-{\mathbf {k}}]\\&= g[(0,1),(1,0)]g[(0,1),(0,2)]+g[(0,1),(0,1)]g[(0,1),(1,1)]\\&\quad +g[(0,1),(1,1)]g[(0,1),(0,1)]+g[(0,1),(0,2)]g[(0,1),(1,0)]=2g_{2,4}=2\overline{b}. \end{aligned}$$

Finally, the leading two rows of ${\mathbf {H}}$ determine a third order normal form for the system of ordinary differential equations:

$$\left\{ \begin{array}{l} \dfrac{dy_{1}}{dt}=i y_1 + p y_{1} y_{2}+ q {y}_{1}^{2}{y}_{2} + O\left( \Vert {\mathbf {Y}}\Vert ^4\right) ,\\ \dfrac{dy_{2}}{dt}=-i y_2 + \overline{p} y_{1} {y}_{2} + \overline{q} y_{1} y_{2}^{2} + O\left( \Vert {\mathbf {Y}}\Vert ^4\right) . \end{array} \right.$$

## Results and discussion

The algorithm presented here for constructing the normal form and normalizing transformation is based on Carleman linearization of differential equations (4) to the form (6) by (5). This algorithm permits us to write the normalizing transformation in matrix form (7) by (9), (3) and the normal form of the differential system by (10), (5) in the sequence (8).

Performing this algorithm using computers presents no difficulties. The algorithm provides explicit recursive formulas for the coefficients of the normal form and the corresponding transformation.

## Conclusions

In this paper, we apply Carleman linearization to the problem of constructing the Poincaré normal form for non-autonomous differential equations with quasi-periodic coefficients. We obtain a recursive algorithm for computing the normalizing transformation and the normal form of the differential system. We also provide a rigorous proof of the validity of the matrix representation of the normalization.
